# MASTICATORY FUNCTION OF OBESE CANDIDATES TO BARIATRIC SURGERY FROM DISTINCT SOCIOECONOMIC CLASSES

**DOI:** 10.1590/0102-6720201600S10014

**Published:** 2016

**Authors:** Celso Roberto PASSERI, Jacira Alves Caracik de Camargo ANDRADE, Karla Thaíza TOMAL, Eduardo Marcucci PRACUCHO, Livia Paschoalino de CAMPOS, Silvia Helena de Carvalho SALES-PERES

**Affiliations:** 1 Bariatric Surgery Service, Amaral Carvalho Hospital, Jau, SP;; 2 Bauru School of Dentistry, University of São Paulo, Bauru, SP;; 3 Botucatu Faculty of Medicine, Universidade Paulista, Botucatu, SP, Brazil.

**Keywords:** Bariatric surgery, Epidemiologic Factors, Hematologic tests, Obesity, Oral health

## Abstract

**Background:**

Obesity and metabolic syndrome can be labeled as worldwide outbreak; thus, both have led to serious public health problem. Oral health can be worsened by both, obesity and metabolic syndrome. Tooth loss harms masticatory function, essential status to whom will be submitted to bariatric surgery.

**Aim:**

Assess masticatory function of obese candidates to bariatric surgery, who belong to distinct socioeconomic class range, in order to recognize hazard factors and the bias of socioeconomic factor in this context.

**Methods:**

Observational cross-section study, with samples comprised by two groups of patients, with distinct socioeconomic class range, one of them belonging to public health system (SUSG) and the other to private clinic (CPG), candidates to bariatric surgery. Were assessed anthropometric data, comorbidities and medicines usage, blood tests, habits and the number of dental functional units.

**Results:**

The groups SUSG and CPG were homogeneous taking into account gender (p=0,890) and age range (p=0,170). The number of dental functional units was higher in the private group (p<0.001). The impaired masticatory function was rather present among public group (p<0.001) and female gender (p<0,001). Regarded as blood tests, fasting glucose was higher in female in SUSG (p<0,001). The following hazard factors have corroborated to have patients rated as impaired masticatory function: belong to public service (OR: 8.420, p=0.003), higher age (OR: 1.186, p<0.001), female gender (OR: 0.153, p=0.029), diabetes mellitus (OR: 2.545, p=0.045) and smokers (OR: 2.951, p=0.043).

**Conclusion:**

The general health and masticatory function of female SUSG were worse, highlighting the socioeconomic condition as hazard factor.

## RESUMO

**Racional:**

Obesidade e síndrome metabólica são graves problemas de saúde pública, com características de epidemia mundial. A saúde bucal é agravada por ambas as condições. Perda dentária prejudica função mastigatória, condição essencial para o paciente que será submetido à cirurgia bariátrica.

**Objetivo:**

Avaliar a função mastigatória de pacientes obesos candidatos à cirurgia bariátrica pertencentes a dois serviços de saúde com padrões socioeconômicos distintos, afim de identificar fatores de risco e a influência do fator socioeconômico nesta condição.

**Métodos:**

Estudo observacional transversal, com amostra constituída por dois grupos de pacientes obesos, com condições socioeconômicas distintas, um pertencente ao sistema público de saúde (GSUS) e outro à clínica privada (GCP), candidatos à cirurgia bariátrica. Foram analisados dados antropométricos, comorbidades e uso de medicamentos para seu controle, exames laboratoriais, hábitos e o número de unidades funcionais dentárias presentes.

**Resultados:**

Os grupos GSUS e GCP foram homogêneos quanto ao gênero (p=0,890) e faixa etária (p=0,170). A média de unidades funcionais dentárias foi maior no grupo privado (p<0,001). A função mastigatória prejudicada foi mais presente no grupo GSUS e no gênero feminino (p<0,001). Quanto aos exames laboratoriais: glicemia de jejum esteve mais alterada em mulheres do GSUS (p<0,001). Foram fatores de risco independentes para ter função mastigatória prejudicada: ter origem no serviço público de saúde (OR: 8,420 - p=0,003), maior idade (OR: 1,186 - p<0,001), ser do gênero feminino (OR: 0,153 - p=0,029), portador de diabete melito (OR: 2,545 - p=0,045) e tabagista (OR: 2,951 - p=0,043).

**Conclusão:**

A saúde geral e função mastigatória de mulheres do GSUS foram piores, ressaltando a condição socioeconômica como fator de risco.

## INTRODUCTION

Obesity and metabolic syndrome are considered serious public health problems worldwide and 2.8% of the world Gross Domestic Product is spent due to excessive fat^8^. In Brazil, 52.5% of the population is overweight, indicating the urge for a more intense investment inside the health system in the decades to come^22^. This condition can trigger a myriad of related diseases, being responsible for patient's autonomy loss and one of the most important death causes around the world^3^.

Due to the lack of efficient long-term clinic treatments against overweight and comorbidity control, bariatric surgery has become a safe and effective alternative^23^.

Series of changes in oral health of obese patients have been released in literature, such as: caries, periodontal disease, xerostomy, dental loss and dental abrasion, showing that there is a strict relation between obesity, metabolic syndrome and oral health, as well as the bariatric surgeries effects on it^4^ ^,^ ^6^ ^,^ ^27^ ^,^ ^29^ ^,^ ^30^. The main reasons for dental loss in non-obese population are periodontal disease and caries^10^ ^,^ ^20^. Obesity and metabolic syndrome raised the incidence and speed up the evolution of these oral diseases^4,27,29,30.^

Masticatory function has great importance to obese who are candidates for bariatric surgery. Pre-operative protocols evince the need for changes on alimentary habits, including an effective chewing that allows better adaptation to the future new anatomic condition of the alimentary canal. These actions can avoid food deviation, which can lead to a decrease in the patients' wellness^7^ ^,^ ^9^ ^,^ ^15^ ^,^ ^26^.

Efficient masticatory function is strictly related to both quantity of existing functional dental unities and masticatory cycle^28^. Functional unity means the tooth that has an antagonist, allowing mastication. It has been shown that the minimum number of eight masticatory functional unities is important for keeping of the efficient masticatory function, allowing the patient's adaptation to the increase of masticatory dynamics^13^. These concepts show the requirement for identification of people who suffer from impaired masticatory function in bariatric surgical programs.

Socioeconomic condition influences both oral health and obesity^5^ ^,^ ^21^ ^,34^. In order to assess this variable's effect on obese patients who are bariatric surgery candidates, different groups were investigated: on one side, people with better socioeconomic conditions and higher access to health care, belonging to the private system; on the other side, people with a bad socioeconomic condition and lower access to health care, belonging to the public health system.

This study's goal was to verify if there is a difference between both groups regarding the amount of dental unities and consequent impairment of the masticatory function. It also aimed to identify which epidemiological, anthropometric and laboratory parameters correlate to this possible difference. 

## METHODS

The Hospital Amaral Carvalho's Ethics Committee in Research, sentence number 1,005,103, has approved this study.

A cross-sectional observational study was accomplished on applicant patients to bariatric surgery, attended in a row, between June/2010 and December/2014. These patients have completed the pre-operative preparation, attended by the same multi-professional team, however being from different health services and with distinct socioeconomic standards. One group belonged to private practice (PCG) with average monthly income over six minimum salaries. The other group belonged to the public system (SUSG) with average monthly income of less than two minimum salaries.

Electronic charts were assessed and data regarding socio-demographic, anthropometric, laboratory, comorbidities, medicine use, habits and number of functional units were collected.

The anthropometric data assessed were: age rated in three ranges (17 to 29, 30 to 45 and over 45); gender and marital status, also rated in three categories (single, married/in a stable union, widower/divorced); weight, height and body mass index (BMI) categorized in three ranges (35 to 39.9, 40 to 49.9 and over 50 kg/m^2^).

Regarding laboratory exams data, only values outside the normal range were taken into account, according to the standard settled by the laboratory responsible for the examination in both groups. Were assessed hemoglobin, hematocrit, ferritin, total proteins, albumin, total cholesterol, triglycerides and fasting glucose.

The presence of obesity related comorbidities was studied, such as arterial hypertension, diabetes mellitus, obstructive sleep apnea, arthropathy and metabolic syndrome. It was also studied the number of daily prescribed medicine according to its pharmacological class, in order to comorbidity control (antihypertensive, oral hypoglycemic, insulin, statins, diuretics, vasodilators and antiplatelet).

 Patients who either made use of tobacco in the treatment meantime or who has smoked 10 cigarettes per day throughout 10 years were labeled as smokers. Alcoholism was evaluated on the AUDIT^2^ scale (The Alcohol Use Disorders Identification Test), being considered present if the score is 16 or over, and absent if the score is 15 or under.

The number of functional dental units was assessed in a clinic exam accomplished by the bariatric surgeon. Godlewski's criteria^13^ were used, which considers as efficient masticatory function carrier patients with eight or more functional dental units. Patients with seven or less units were considered inefficient masticatory function carriers.

Ultimately, it was assessed the period (in days) between the first medical appointment and the bariatric surgery's accomplishment date, being rated in three ranges: between 0 and 180; between 181 and 365; and over 365 days.

### Statistical analysis

Was accomplished using IBM SPSS 19 program (Chicago - USA). Regarding continuous variables, the data's descriptive analysis was done through the average's calculation and its respective pattern deviations. The analysis regarding categorical variable was done through presentation of absolute and relative frequencies. In order to verify the distributions' normality, Kolmogorov-Smirnov's test was applied. Comparisons between the averages were accomplished using analysis of variance test (ANOVA) for continuous variables. Chi-square test was used for the assessment of categorical variables. The meaningfulness level applied was of 5% (p<0.05). The study regarding the relation between anthropometric, clinics, habits, origins service variables and the number of functional dental units was accomplished by the multiple logistic regression. In order to assess which of these variables independently interfered with the masticatory function, the multiple logistic regression was supplemented with gaps ranging until 95% trust for the risk ratios (OR)^14^. The sample was subdivided into four groups according to the origin service (SUSG or PCG) and the masticatory condition (impaired or efficient) in order to correlate them with gender, age range, marital status and waiting time for the surgery. Goodman's correlation test was applied^12^ with a meaningfulness level of 5%. The association between altered laboratorial tests, origin services and number of functional dental units was accomplished using Goodman's association test. It was also complemented with multiple comparisons between and within multinomial populations, considering 5% of meaningfulness level^12^.

## RESULTS

The private system (PCG) was composed by 141 patients [male: 25 (17.7%) and female: 116 (82.3%)], while the public system (SUSG) was composed by 267 patients [male: 45 (16.9%) and female: 222 (83.1%)]. Both systems were homogenous regarding gender (p=0.890) and average age (p=0.174), with predominance of female gender in both of 4.8:1 (p<0.001). The age range between 17 and 29 years old was predominant in female PPG (p=0.037); the rest had similar distribution between services.

Regarding marital status, the category "single" was predominant in female SUSG (p=0.009). The rest was distributed evenly.

The average weight, overweight and BMI were higher in SUSG in both genders (male and female: p<0.001). Inside PCG and SUSG, male gender has shown higher indexes than female (p<0.001).

The average number of dental functional units in PCG, in both genders, showed the highest average compared to SUSG (p<0.001). In SUSG, male gender showed higher average compared to female gender (p=0.008). When comparing both groups regarding impaired masticatory function (≤7 functional units) and efficient masticatory function (≥8 functional units), the impaired function was more present in female SUSG (p<0.001) and the efficient one in female PCG (p<0.001). Inside SUSG, the male gender showed predominance of efficient masticatory function when compared to female gender (p=0.036).

Smoking didn't show different incidence between both services, while alcoholism showed predominance in SUSG when compared to PCG (0.014). Male gender showed higher alcoholism rate in both services [PCG (p=0.031) and SUSG (p<0.001)].

The average number of patients who made use of medicine showed homogenous distribution in both services and genders. However, the average of medicine used by pharmacological class was higher in female SUSG (p<0.001).

Laboratorial tests didn't show difference between both services regarding hemoglobin, hematocrit, ferritin, total cholesterol and triglycerides. Total proteins and albumin were more frequently outside standard values in PCG (p=0.045 and p=0.040 respectively). Fasting glucose was more altered in female SUSG (p<0.001), while in PCG it was more altered in male gender than female (p=0.012).

Comorbidities presence was higher in female SUSG: arterial hypertension (p=0.005), diabetes mellitus (p=0.006), sleep apnea (p=0.004) and metabolic syndrome (p<0.001). Inside the private system (PCG), the male gender showed higher frequency of arterial hypertension (p=0.022), sleep apnea (p=0.003) and metabolic syndrome (p=0.003). Arthropathy was more frequent in PCG when compared to SUSG in both genders (male: p=0.042 and female: p=0.007).

 The average period between the first medical appointment and the surgery's accomplishment date was higher in female SUSG (p<0.001). Regarding the period range, the one between 0 and 180 days was predominant in female PCG (p<0.001) and the one over 365 days was predominant in female SUSG (<0.001).

This sample features that: being from public service (p=0.003), advanced age (p<0.001), being female (p=0.029), suffer from diabetes (p=0.045) and being smoker (p=0.043) were independent risk factors that can trigger impaired masticatory function.

It is able to assert that the female gender was predominant in all four groups. Patients with efficient masticatory function were more frequent in PCG in both genders. Impaired masticatory function, both in PCG and in SUSG, was predominant in over 45 years old range. Within the range between 30 and 45, public system patients' percentage was higher when compared to private system patients also regarding impaired masticatory function. In the age range between 17 and 29 years old, no patients with impaired masticatory function were found. When compared public and private systems, no statistic significance between the groups regarding marital status was found. Single people with efficient masticatory function are predominant in both private and public systems. Married/in a stable union people with efficient masticatory function were predominant in PCG, while in SUSG were predominant with impaired masticatory function. When comparing the waiting time between the first medical appointment and the surgery's date in SUSG, over 365 days range was predominant between patients with impaired masticatory function. In PCG, patients with efficient masticatory function were more frequent in the range between 0 and 180 days.

**TABLE 1:**
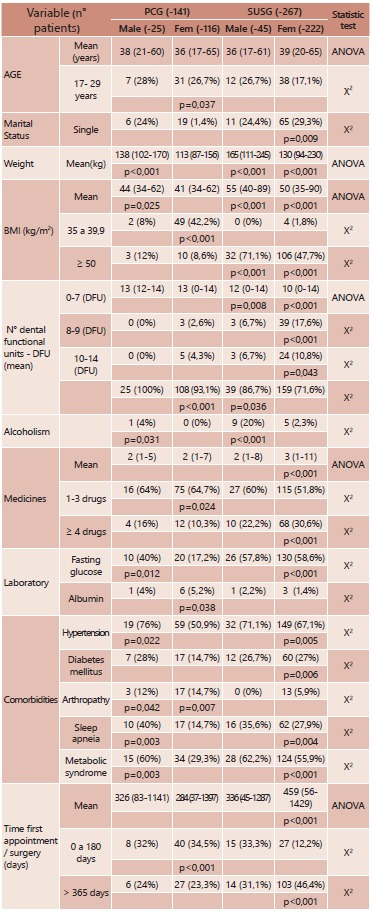
Distribution of sociodemographic, anthropometric, number of functional dental unities, laboratorial tests, habits, medicines in usage, comorbidities, period between first appointment and surgery date variable distribution according to origin services and gender

χ2 = Chi-Square ; p<0,05

**TABLE 2:** Multiple logistic regression of the functional dental units quantity taking into account sociodemographic, anthropometric, habits and clinical data

Variable	Logistic regression coefficient (EP)	Odds Ratio (OR)	p	CI (OR)
Regression constant	- 9.939 (2.477)	-	-	-
Origen service (Public/Private)	2.131 (0.718)	8.420	0.003	(2.062; 34.385)
Age (years)	0.170 (0.0295)	1.186	<0.001	(1.119; 1.256)
Gender (male/female)	- 1.878 (0.859)	0.153	0.029	(0.0284; 0.823)
Waiting time for surgery (days)	0.00143 (0.000666)	1.001	0.032	(1.00; 1.003)
BMI (kg/m^2^)	- 0.0967 (0.0987)	0.908	0.327	(0.748; 1.102)
Overweight (kg)	0.0421 (0.0384)	1.043	0.272	(0.967; 1.124)
Hypertension (Absent / Present)	- 0.303 (0.551)	0.739	0.583	(0.251; 2.177)
Obstructive sleep apnea ((Absent / Present)	- 0.276 (0.478)	0.759	0.564	(0.297; 1.938)
Diabetes Mellitus (Absent / Present)	0.934 (0,466)	2.545	0.045	(1.021; 6.342)
Arthropaties (Absent / Present)	- 0.253 (0.828)	0.777	0.760	(0.153; 3.933)
Smoking (Absent / Present)	1.082 (0,535)	2.951	0.043	(1.035; 8.414)
Alcoholism (Absent / Present)	0.758 (1.067)	2.134	0.477	(0.264; 17.280)
Number of medicines usage	0.0187 (0.0992)	1.019	0.851	(0.839; 1.238)

Hosner & Lemeshow; confidence interval 95% for OR

**TABLE 3:** Impaired and efficient masticatory function percentage regarding the variables: gender, age range, marital status and waiting time for the surgery, separating both SUSG and PCG.

Variable	Origen service x masticatory function			
	Private impaired (3 cases)	Private efficient (138 cases)	Public impaired (42 cases)	Public efficient (225 cases)
Gender Male Female	0 100 ¶	18,1 # 81,9¶ #	7,1 92,9 ¶	18,7 81,3 ¶
Age ranges (years) (1) 17 a 29 (2) 30 a 45 (3) > 45	0 0 100 ¤	27,9 # 50 ∆ 22,1 #	0 26,2 v ∆ 73,8 v ∆	22,2 # 56,9 # ∆ 20,9 #
Marital Status Single Marriage /Cohabiting Split / Div / Widow	0 66,7 33,3	27 # ∂ 65,7 ∞ 7,3 x	14,3 64,3 ∞ 21,4	31,1 ∂ 60,4 8,4 x
Waiting time (days) ≤ 180 181 a 365 > 365	0 66,7 33,3	34,8 # 41,3 23,9	4,8 26,2 69 ∏	18,7 #v¢ 41,8 39,6 #v

Goodman association test p< 0.05;¶ male x female; # impaired x efficient; v private x public; ¤ age range 3 x the others; ∆ age range 1 x age range 2; ∂ singles x marriages and cohabiting, divorced and widows; ∶ marriages and cohabiting x splits, divorced and widows; ∞ marriages and cohabiting x splits, divorced and widows; ∴ > 365 x the others; ∏ ≤ 180 x the others

## DISCUSSION

The results achieved concluded that the obese applicants for bariatric surgery with rather graver cases of dental loss in the first appointment and consequently worst masticatory function showed the following features: from SUSG, female gender, advanced age, diabetes mellitus carrier and smoker. This condition led to a more lingering period from the first medical appointment until the surgery's accomplishment date. These facts evince that socioeconomic condition is strictly associated with the patient's systemic and oral health.

The female gender was predominant in both private and public systems. The research showed difference in the results of several weight loss treatments between the male and female genders, the first one being more efficient both in weight loss and maintenance after the loss^25^. Another prospective longitudinal study showed the difference between male and female genders regarding the evolutionary pattern of weight gain throughout the following decades after the surgery was undergone, with progression of overweight and obesity. BMI increase and abdominal circumference enlargement were higher in young adults when compared to elders in both genders, being even more intense on women. After the sixth decade, this gain ceased in men and maintained progressive in women. Besides that, central obesity was more prevalent in female gender, what increases the risk of comorbidities^16^. In Brazil, the differences in the association of dental loss with obesity among old adults were shown, where the incidence of central obesity is higher than general obesity^24^. There is a relation between central obesity and edentulousness, while this relation doesn't occur in general obesity. Moreover, it has been proven that edentulous women have a higher chance of suffering from obesity when compared to men. These data indicate the importance of searching for bariatric surgery and the reasons for the low conditions of general and oral health in female patients.

It was established in the study that the average age in both SUSG and PCG were similar, being predominant in the fourth decade. This fact can be explained using some clinic observations done during the medical appointments: unsuccessful attempts to clinic treatments, obesity gained throughout pregnancies, concluded reproductive cycle in women and onset of comorbidities clinical manifestations. All these features can lead the patient to try the bariatric surgery. A study released showed that the main reason for pursuing weight loss by American women over 40 was corporal image (nine times more than clinical health). Many of these patients had, in their previous history, frustrated attempts to clinical treatment against obesity, triggering the search for surgical treatment within intermediate age range. The main factor that unleashed obesity was consecutive pregnancies^1^.

The categorization by age range showed that the youngest group (17 to 29 years old) was prevalent in female PCG (p=0.037). The psychosocial inclusion and the willingness to have a better corporal image in this age range are factors that can explain the young women's interest in bariatric surgery^11^.

Age was an independent risk factor regarding impaired masticatory function. Every year the patient ages, the possibility of carrying impaired masticatory function grows 1.186 times (p<0.001). No patients carrying impaired masticatory function were found in both SUSG and PCG within the age range between 17 and 29 years old.

Regarding marital status, the group married/in a stable union was prevalent in the sample, with 64% of total. The correlation between marital status and masticatory condition showed that single patients have a higher frequency of efficient masticatory function. Age range could have biased this data, since the single group is concomitant with the youngest population of the sample.

The initial weight, BMI and overweight averages were higher in SUSG than PCG (p<0.05). Many studies show the paradox between bad socioeconomic condition and the higher obesity incidence. The phenomenon poverty/obesity has been sorely studied and some data have been released in order to attempt to explain it. Both the easy access to industrialized food, with high calorific values and low prices, and expensive cost of doing physical exercises (gym and sport goods) could be implicated in this phenomenon^31^.

Level II obesity carriers were prevalent in female PCG, evincing a more premature search for the surgery. The psychosocial pressure and the easy access to healthcare can maybe explain this fact. Meanwhile, super-obesity was predominant in SUSG in both genders. This data confirms previous studies that relate bad socioeconomic conditions to obesity and harder access to health system^31^.

The correlation between BMI, overweight and masticatory function condition hasn't reached any statistic meaning in this sample. The relation between obesity and general and oral health condition has already been showed in previous studies^27^ ^,^ ^29^. This fact can be explained by the inexistence of patients in the standard weight range, it means the unanimous presence of obesity patients in the sample who are exposed to the same risk condition.

Efficient masticatory condition is primordial to a patient who will be undergone to bariatric surgery^7^ ^,^ ^9^. When all the patients' dentition was assessed, the average number of function dental unities was higher in PCG than SUSG in both genders (p<0.001). When assessed the number of patients who carries impaired masticatory function, this group was higher in female SUSG, showing a worse oral health condition. When assessed the number of patients with efficient masticatory function, this group was higher in PCG, without gender distinction. Studies released showed the existence of a strict interaction between oral health (mainly periodontal disease) and obesity, getting even worse when associated with metabolic syndrome^4^ ^,^ ^29^ ^,^ ^30^. The main etiologies that favor dental loss in adults are periodontal disease and dental caries^20^. The female SUSG showed higher obesity level when compared to female PCG, besides higher number of patients who suffer from associated comorbidities, such as diabetes mellitus and metabolic syndrome. The number of functional dental unities loss was also higher, evincing worse systemic and oral health. This data was corroborated to similar findings in other studies, highlighting the strict relation between systemic and oral health conditions^19^.

Since female SUSG has fewer number of functional dental units e and more patients who carry diabetes mellitus (p=0.006) and metabolic syndrome (p<0.001), it is wondered if obesity and metabolic syndrome are triggered by oral health condition or vice-versa. Longitudinal studies must be conducted in order to enlighten this relation.

The habit of smoking showed a homogenous distribution between the services (SUSG and PCG) and genders. The harmful aspect of this habit in periodontal disease is already known. Periodontal disease receives multifactorial influence in its evolution, including access to the treatment, nourishing habits and systemic health conditions. In this sample, for instance, diabetes mellitus and metabolic syndrome were more present in female PSG, being the habit of smoking only an adding factor in periodontal disease's evolutionary process^18^.

Furthermore, the habit of smoking was an independent risk factor in the sample, when smokers had a 2.9 times higher probability of carrying impaired masticatory function. This variable has been established in literature as negative influence factor in the evolution of oral pathologies, therefore acting as contributor to functional dental unities loss^18^.

Alcoholism was higher in SUSG when assessed in the total sample (p=0.014). It was also higher in male gender both in PCG (p=0.031) and SUSG (p<0.001). Alcoholism didn't reach statistic meaning regarding the risk of impaired masticatory function. Maybe this fact is explained due to the higher incidence of alcoholism in male gender, which is less represented in the total sample, what hinders statistic analysis.

 The laboratory tests assessment showed that unusual fasting glucose dosages were more frequent in female SUSG (p<0.001). The data also showed that the number of patients with unusual fasting glucose dosages was higher in SUSG with impaired mastication (p<0.05). It's known that diabetes mellitus has a strict relation with periodontal disease, which is one of the main causes of dental loss in adults. So it's possible to conclude that the data collected are in consonance with dental and medical literatures^4^. 

The presence of comorbidities wasn't homogenous neither in the origin services nor in the genders. Arterial hypertension (p=0.005), diabetes mellitus (p=0.006), obstructive sleep apnea (p=0.004) and metabolic syndrome (p=0.001) were prevalent in female SUSG. All these diseases are strictly related to the obesity level and the evolution time of the patient's obese condition^30^. Diabetes mellitus was the only comorbidity that had statistic meaningfulness as independent risk factor to impaired masticatory function (p=0.045). As it was mentioned before, this comorbidity has an intimate relation with periodontal disease, what triggers dental loss in adults^4^.

The carriage of arterial hypertension (p=0.583), obstructive sleep apnea (p=0.564) and arthropathy (p=0,760) wasn't an independent risk factor with statistic meaning to impaired masticatory function.

The average waiting time from the first medical appointment until the surgery accomplishment date was higher in SUSG (p<0.001), which also had higher number of patients who lingered more than 365 days until the surgery (p<0.001). The surgeries accomplished until 180 days were higher in female PCG (p<0.001). All analysis had done so far show consistency in the information. Both bariatric surgery services (SUSG and PCG) are conducted by the same multi-professional team and follow the same pre-operative norms, where no scheduling restriction according to the origin service is done. It's important to highlight that, in both services, no surgery is accomplished before all clinic alterations detected are treated. Adding to that, the patients who carry impaired masticatory function are advised to amend its condition.

The waiting time in the range above 365 days was prevalent in patients from SUSG with impaired masticatory function, according to the association test. Patients from PCG with efficient masticatory function have prevalence of the time range until 180 days.

The patients from SUSG showed the risk 8.4 times higher of carrying impaired masticatory function when compared to PCG patients. The results reached in this study corroborates to literature data, which show the influence of socioeconomic condition in systemic and oral health.

Some limitations of methodological genus must be mentioned. The first one is guided in the study's type, which was observational transversal, what limits the facts triggering analysis. The second one relates to laboratorial tests of cholesterol fraction in patients from SUSG, which in sake of this test's high cost, only was compared total values. That factor limited one of the parameters analysis (HDL cholesterol dosage) used in the metabolic syndrome's diagnosis.

Prospective studies should be accomplished in order to measure the impact of masticatory function rehabilitation in bariatric surgery results, regarding weight loss, comorbidities control and wellness.

## CONCLUSION

The variables which showed relation to impaired masticatory function were: origin service, age, gender, diabetes mellitus and smoking, all of them triggering off a lingering waiting time for the surgery. This finding indicates that women from public service (with worse socioeconomic condition), with advanced age, smokers and diabetes mellitus carriers demand more attention coming from multi-professional teams, regarding systemic and oral health. If this measure is taken in the beginning of the surgery preparation, it could avoid lingering waiting time for its accomplishment. 

## References

[1] Anderson LA, Eyler AA, Galuska DA, Brown DR, Brownson RC (2002). Relationship of satisfaction with body size and trying to lose weight
in a national survey of overweight and obese women aged 40 and older, United
States. Prev Med.

[2] Babor TF, Higgins-Biddle JC, Saunders JB, Monteiro MG (2001). The alcohol use disorders identification test. Guidelines for use in primary
care. Second Edition.

[3] Belle SH, Chapman W, Courcoulas AP, Flum DR, Gagner M, Inabnet WB (2008). Relationship of body mass index with demographic and clinical
characteristics in the Longitudinal Assessment of Bariatric Surgery
(LABS). Surg Obes Relat Dis.

[4] Benguigui C, Bongard V, Ruidavets JB, Chamontin B, Sixou M, Ferrieres J (2010). Metabolic syndrome, insulin resistance, and periodontitis a
cross-sectional study in a middle-aged French population. J Clin Periodontol.

[5] Chalub L, Borges CM, Ferreira RC, Haddad JPA, Ferreira EFE, Vargas AMD (2014). Association between social determinants of health and functional
dentition in 35-year-old to 44-year-old Brazilian adults a population-based
analytical study. Community Dent Oral Epidemiol.

[6] De Moura-Grec PG, Yamashita JM, Marsicano JA, Ceneviva R, de Souza Leite CV, de Brito GB (2014). Impact of bariatric surgery on oral health conditions 6-months cohort
study. Int Dent J.

[7] Di Vetta V, Kraytem A, Giusti V. (2008). Gastric bypass: management of complications and food
tolerance. Rev Med Suisse.

[8] Dobbs R, Sawers C, Thompson F, Manyika J, Woetzel J, Child P, McKenna S, Spatharou A (2014). Overcoming obesity:?An initial economic analysis.

[9] Endevelt R, Ben-Assuli O, Klain E, Zelber-Sagi S (2013). The role of dietician follow-up in the success of bariatric
surgery. Surg Obes Relat Dis.

[10] Fukuda H, Saito T, Mizuta M, Moromugi S, Ishimatsu T, Nishikado S (2013). Chewing number is related to incremental increases in body weight from
20years of age in Japanese middle-aged adults. Gerodontology.

[11] Furnham A, Dias M, McClelland A (1998). The role of body weight, waist-to-hip ratio, and breast size in
judgments of female attractiveness. Sex Roles.

[12] Goodman LA (1965). On simultaneous confidence intervals for multinomial
proportions. Technometrics.

[13] Godlewski AE, Veyrune JL, Nicolas E, Ciangura CA, Chaussain CC, Czernichow S (2011). Effect of Dental Status on Changes in Mastication in Patients with
Obesity following Bariatric Surgery. Plos One.

[14] Hosmer DW, Lemeshow S (2000). Applied logistic regression.

[15] Hyvarinen K, Salminen A, Salomaa V, Pussinen PJ (2015). Systemic exposure to a common periodontal pathogen and missing teeth
are associated with metabolic syndrome. Acta Diabetol.

[16] Kimokoti RW, Newby PK, Gona P, Zhu L, O'Malley CM, Guzman JP (2012). Patterns of weight change and progression to owerweight and obesity
differ in men and women: implications for research and
interventions. Public Health Nutr.

[17] Martin-Rodriguez E, Guillen-Grima F, Martí A, Brugos-Larumbe A (2015). Comorbidity associated with obesity in a large population: The APNA
study. Obes Res Clin Pract.

[18] Morse DE, Avlund K, Christensen LB, Fiehn NE, Molbo D, Holmstrup P (2014). Smoking and Drinking as Risk Indicators for Tooth Loss in Middle-Aged
Danes. J Aging Health.

[19] Oluwagbemigun K, Dietrich T, Pischon N, Bergmann M, Boeing H (2015). Association between Number of Teeth and Chronic Systemic Diseases A
Cohort Study Followed for 13 Years. PLoS One.

[20] Phipps KR, Stevens VJ (1995). Relative contribution of caries and periodontal disease in adult tooth
loss for an HMO dental population. J Public Health Dent.

[21] Pilotto LM, Celeste RK, Faerstein E, de Slavutzky SMB (2014). Association between tooth loss and overweight/obesity among Brazilian
adults the Pro-Saude Study. Braz Oral Res.

[22] Portal Brasil, Ministério da Saúde (2015). Metade dos brasileiros está com excesso de peso.

[23] Schauer PR, Bhatt DL, Kirwan JP, Wolski K, Brethauer SA, Navaneethan SD (2014). Bariatric Surgery versus Intensive Medical Therapy for Diabetes-3-Year
Outcomes. N Engl J Med.

[24] Singh A, Peres MA, Peres KG, Bernardo CO, Xavier A, D'Orsi E (2015). Gender differences in the association between tooth loss and obesity
among older adults in Brazil. Rev Saude Pública.

[25] Stroebele-Benschop N, Damms-Machado A, Milan FMP, Hilzendegen C, Bischoff SC (2013). Gender differences in the outcome of obesity treatments and weight
loss maintenance - A systematic review. J Obes Weight Loss Ther.

[26] Stumpf MAM, Rodrigues MRS, Kluthcovsky ACGC, Travalini F, Milléo FQ (2015). Análise da tolerância alimentar em pacientes submetidos à cirurgia
bariátrica através do questionário quality of alimentation. ABCD, arq. bras. cir. dig.

[27] Suvan J, D'Aiuto F, Moles DR, Petrie A, Donos N (2011). Association between overweight/obesity and periodontitis in adults A
systematic review. Obes Rev.

[28] Ueno M, Yanagisawa T, Shinada K, Ohara S, Kawaguchi Y (2010). Category of functional tooth units in relation to the number of teeth
and masticatory ability in Japanese adults. Clin Oral Investig.

[29] Yamashita JM, Moura-Grec PG, Freitas AR, Sales-Peres A, Groppo FC, Ceneviva R (2015). Assessment of Oral Conditions and Quality of Life in Morbid Obese and
Normal Weight Individuals: A Cross-Sectional Study. PLoS One.

[30] Zhu Y, Hollis JH (2015). Associations between the number of natural teeth and metabolic
syndrome in adults. J Clin Periodontol.

[31] Zukiewicz-Sobczak W, Wroblewska P, Zwolinski J, Chmielewska-Badora J, Adamczuk P, Krasowska E (2014). Obesity and poverty paradox in developed countries. Ann Agric Environ Med.

